# Putative Roles of SETBP1 Dosage on the SET Oncogene to Affect Brain Development

**DOI:** 10.3389/fnins.2022.813430

**Published:** 2022-05-24

**Authors:** Lilit Antonyan, Carl Ernst

**Affiliations:** Department of Human Genetics, McGill University, Montreal, QC, Canada

**Keywords:** neurodevelopment, SETBP1, Schinzel–Giedion syndrome, disease modeling, gene

## Abstract

Mutations in *SET BINDING PROTEIN 1* (*SETBP1*) cause two different clinically distinguishable diseases called Schinzel–Giedion syndrome (SGS) or *SETBP1* deficiency syndrome (SDD). Both disorders are disorders of protein dosage, where SGS is caused by decreased rate of protein breakdown due to mutations in a proteosome targeting domain, and SDD is caused by heterozygous loss-of-function mutations leading to haploinsufficiency. While phenotypes of affected individuals support a role for SETBP1 in brain development, little is known about the mechanisms that might underlie this. The binding partner which gave SETBP1 its name is SET and there is extensive literature on this important oncogene in non-neural tissues. Here we describe different molecular complexes in which SET is involved as well as the role of these complexes in brain development. Based on this information, we postulate how SETBP1 protein dosage might influence these SET-containing molecular pathways and affect brain development. We examine the roles of SET and SETBP1 in acetylation inhibition, phosphatase activity, DNA repair, and cell cycle control. This work provides testable hypotheses for how altered SETBP1 protein dosage affects brain development.

## Introduction

*SET BINDING PROTEIN 1* (*SETBP1*) germline mutations cause disorders of varying severity, but how *SETBP1* mutations lead to disease is not known. There are a substantial number of studies on the molecular function of the protein SET (von Lindern et al., 1992b; Bayarkhangai et al., 2018) in non-brain tissue, a protein with which SETBP1 is known to interact. Examining the actions of SET in any tissue type might provide some insight into the role of SETBP1 in brain since intrinsic cell activators and repressors can be used in different ways across tissue types. This does not rule out other roles for SETBP1 independent from SET which we do address in a section of this review; however, we have chosen to condition our analysis of SETBP1 function on SET, because of the interaction between these two proteins (Minakuchi et al., 2001) and the large body of work that pertains to SET.

The purpose of this review is to describe potentially relevant mechanistic studies from SET, then to assess whether mechanisms regulated by SET in non-neural tissue could play a role in brain development. We will integrate these two components to postulate how mutations in *SETBP1* could affect brain development and lead to disease phenotypes associated with *SETBP1* mutations. Our hope is to generate testable hypotheses targeting specific molecular systems that may be important for the etiology underlying neurodevelopmental diseases caused by mutations in *SETBP1*.

## SETBP1 Protein Structure

**FIGURE 1:**
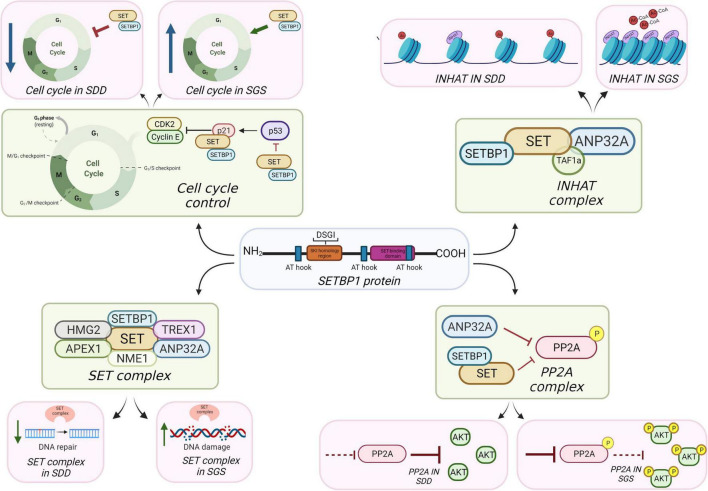
Potential mechanisms of SETBP1 action in brain.

SETBP1 has molecular mass of ∼170 kDa (UniProt:Q9Y6X0) and is found in most tissues. SETBP1 was first termed SEB (Minakuchi et al., 2001) when a yeast two-hybrid screen was performed for binding partners of the important tumor suppressor SET (Minakuchi et al., 2001). In addition to the SET-binding domain, SETBP1 has a SKI homology region (Wilson et al., 2004), three nuclear localization signal (NLS) motifs, three adenine-thymine (AT) hook domains (Coccaro et al., 2017), and six PEST domains (sequences associated with proteins that have a short intracellular half-life) (Figure 1; Rogers et al., 1986). Its three NLS motifs and three AT-hooks suggest that its localization and functions might be primarily nuclear (Minakuchi et al., 2001; Cristobal et al., 2010; Nguyen et al., 2016) and two of the three NLS motifs are found within the SET-binding region. The SKI-homology domain gets its name from the ∼36% homology of this region with the middle region and dimerization domain of nuclear oncoprotein SKI (Bonnon and Atanasoski, 2012), so SETBP1 could mimic SKI/SKI homodimer function in TGFb repression (Wu et al., 2002) or may bind SKI itself. The SKI-homology domain of SETBP1 contains a well-defined degron motif (Wu et al., 2003; Meszaros et al., 2017), a sequence of AAs that needs to be recognized for the protein to be degraded by the proteasome. The degron contains the consensus binding region DpSGXXpS/pT, where pS and pT are phosphorylated residues and X is any amino acid. SCF-β-TrCP1 is the substrate recognition subunit of the E3 ubiquitin ligase, which ubiquitinates SETBP1, targeting it for degradation (Piazza et al., 2013; Meszaros et al., 2017).

## Diseases Associated With Germline *SETBP1* Mutations: Schinzel–Giedion Syndrome and *SETBP1* Deficiency Disorder

The discovery and rapid evolution of DNA sequencing technologies over the last two decades have allowed for the mapping of specific mutations associated with rare diseases. Germline mutations in *SETBP1* cause two different disorders: Schinzel–Giedion syndrome (SGS; *OMIM 269150*) and *SETBP1* deficiency disorder (SDD; *OMIM 606078*), where genomic position of the mutations determines the effect on SETBP1 protein leading to one of the two diseases (Hoischen et al., 2010; Filges et al., 2011). Somatic mutations at the identical site as the SGS mutations cause atypical chronic myeloid leukemia (Piazza et al., 2013).

Schinzel–Giedion syndrome is a rare and severe developmental disease characterized by developmental and growth delay, progressive brain atrophy, delayed myelination and progressive atrophy of white matter, distorted neuronal layering, hydronephrosis, hydrocephalus, midface retraction, visual and hearing impairment, severe seizures, neuroepithelial tumors, genital hypoplasia, bone abnormalities, and other congenital malformations (Schinzel and Giedion, 1978; McPherson, 2006; Beschorner et al., 2007; Filges et al., 2011; Acuna-Hidalgo et al., 2017; Coccaro et al., 2017). The symptoms are so severe that children suffering from SGS usually die within the first decade of life (McPherson, 2006). Although SGS was described in 1978 (Schinzel and Giedion, 1978; AlGazali et al., 1990), it wasn’t until 2010 that the association with the *SETBP1* gene was made (Schinzel and Giedion, 1978; Hoischen et al., 2010). SGS occurs due to *de novo* heterozygous missense mutations affecting AAs of the degron motif at AAs 868–871. “Atypical” SGS cases are also known which affect AAs adjacent or close to AAs 868–871, where those cases reported are phenotypically similar to SGS cases but may live past the first decade of life (Acuna-Hidalgo et al., 2017). The reason for disease is thought to be due to increased SETBP1 protein stability (Piazza et al., 2013, 2018; Nguyen et al., 2016; Acuna-Hidalgo et al., 2017) from the mutations in the degron motif, affecting the ability of the ubiquitin ligase to add ubiquitin to signal degradation. For this reason, SGS is likely a disease of too much protein persisting for too long.

*SETBP1* deficiency disorder is characterized by mild to severe developmental delay, distinctive facial features (prominent forehead, inverted triangle face, ptosis, periorbital fullness), seizures, hypoplastic corpus callosum, early life hypotonia, high sociability, and expressive speech delay (Filges et al., 2011; Marseglia et al., 2012; Jansen et al., 2021; Morgan et al., 2021). Mutations that cause SDD are heterozygous deletions or frameshift mutations that lead to the loss of expression of *SETBP1* from one allele, classifying the disease as a loss-of-function (LOF) syndrome (Cody et al., 2007; Buysse et al., 2008; Bouquillon et al., 2011; Filges et al., 2011; Marseglia et al., 2012).

Schinzel–Giedion syndrome and SDD might be considered on a *SETBP1* dosage spectrum. Both have functional SETBP1 protein, but SDD has too little protein due to haploinsufficiency, while SGS SETBP1 protein persists for too long, due to decreased proteasomal breakdown because of the mutated degron motif. Both proteins retain their normal function implying that *SETBP1* dosage may be critical and that cells do not tolerate dosage changes of SETBP1. It is within this “dosage” context that we will assess the different brain systems in which SETBP1 may be important.

## Brain Development and SETBP1 Function

Brain development is a process by which cells are specialized to become cell types of the forebrain, midbrain, hindbrain, and spinal column. Briefly, after the formation of the ecto-, meso-, and endoderm layers, one of these layers, the ectoderm, begins to receive signals to neuralize in a process called neural induction. This process leads to the folding of the epithelial layer of cells and an invagination process that forms the neural tube. Neurulation is the process by which instructive signals, often diffusible morphogens or extracellular proteins from neighboring cells, are received by this neuralized ectoderm to proliferate and differentiate to form the brain and spinal cord along anterior–posterior and dorsal–ventral axes. The posterior neural tube becomes the spinal column while the anterior neural tube balloons out to become forebrain (telencephalon) and midbrain/hindbrain (mesencephalon/diencephalon) structures derived from proliferating cells from the neuroectoderm (Stiles and Jernigan, 2010). To create the future forebrain for example, neurogenesis begins in the telencephalic primordium with the symmetrical expansion of neural stem cells (NSCs) in the ventricular zone (VZ) (Bystron et al., 2008; Kolk and Rakic, 2022). Some NSCs form a dorso-ventrally anchored scaffold and become radial glial cells, which form the migration pathways for subsequently differentiating neurons (Rakic, 1990). As expansion of the NSC pool of cells occurs, NSCs gradually switch into an asymmetrical mode of cell division where one daughter cell remains a NSC and the other differentiates into a neuron (Molnar et al., 2019). As neurogenesis completes, progenitor cells switch first to an astroglial fate, then an oligodendroglial fate.

Schinzel–Giedion syndrome is a multi-system disorder including brain, while SDD seems almost entirely brain specific. To determine the role SETBP1 may have in brain, we review functions of SET and, if available, SETBP1, in other tissues and apply these findings to how SDD and SGS mutations in *SETBP1* may affect brain development. We expect that a major action of SETBP1 is through binding SET, and so look at known molecular actions of SET in any tissue and interpret what may happen in brain with *SETBP1* mutations. SETBP1 is predicted to stabilize SET, so SGS mutations may lead to more persistent SET, while SDD mutations may lead to less stable SET.

If SETBP1 acts through SET, then we expect that mutations in *SET* itself should affect brain development, and this is indeed the case. Mutations in *SET* lead to varying severity of developmental and speech delay, motor impairment, and intellectual disability (Hamdan et al., 2014; Richardson et al., 2018; Stevens et al., 2018). All reported mutations are heterozygous and most likely cause loss of function of *SET*, even when the causative mutations are missense. This could potentially be a condition on the same spectrum as *SETBP1* SDD mutations, which has been described previously (Richardson et al., 2018). There have been no mutations in SET reported which lead to symptoms similar to SGS. It is possible that mutations in SET could affect its interactions with protein complexes associated with intellectual disability (Richardson et al., 2018).

## The Inhibitor of Histone Acetyltransferase Complex

The inhibitor of acetyltransferases (INHAT) complex is composed of SET, TAF1a (a protein isoform of SET that differs in the first 37 amino acids (UniProt Q01105-2) and ANP32A (Seo et al., 2001; Figure 1). The INHAT complex blocks histone acetyltransferase (HAT) activity of p300/CBP and PCAF by binding to histones and sterically blocking them, possibly through the “earmuff domain” of SET, where SET can bind both core histones and double-stranded DNA (dsDNA) (Seo et al., 2001, 2002; Muto et al., 2007). It is not clear whether this INHAT activity is independent from SET’s histone chaperone activity (Gamble et al., 2005; Gamble and Fisher, 2007). Acetylation of histone residues such as H3K9ac and/or H3K27ac function to mark active enhancers or promoters, meaning that RNA transcription is more likely to occur where histones are acetylated and chromatin is decondensed. Blocking acetylation can condense chromatin and make DNA less accessible to activation.

The acetylation of histones is an important component of molecular events in brain development (Contestabile and Sintoni, 2013; Lilja et al., 2013). Histone acetylation and deacetylation forms part of the histone code, where the timing of addition and removal of acetyl groups likely helps to pre-pattern or to drive important developmental functions (Ernst and Jefri, 2021). In the developing brain, blocking the removal of histones inhibits the differentiation of astrocytes and oligodendrocytes (Marin-Husstege et al., 2002; Hsieh et al., 2004). VZ neural progenitor cells which populate the cerebral cortex are capable of giving rise to neurons, astrocytes, and oligodendrocytes (in that order) (Qian et al., 2000), suggesting acetyl groups must be removed after neurogenesis to allow for the switch to gliogenesis (Balasubramaniyan et al., 2006). Neurodevelopmental diseases of histone acetylation such as Rubinstein–Taybi (*OMIM 180849*) further highlight these important functions (Cohen et al., 2020). INHAT may have a role in the neurodevelopmental process, possibly by ensuring some histones are not acetylated prior to neurogenesis.

SETBP1 might (1) bind SET to inhibit its binding with ANP32A, (2) inhibit the processing of SET protein isoforms in INHAT, or conversely, (3) stabilize SET and make a platform for SET to more easily associate with ANP32A. Evidence from non-brain cancer studies suggest that too much SET is associated with hypoacetylation (Almeida et al., 2017), consistent with its role in blocking acetylation, and this could be the case in developing brain cells with SGS *SETBP1* mutations. That is, that SGS is associated with too much inhibition of histone acetylation and SDD may show hyperacetylation. Blocking acetylation, depending on when this occurs in neurodevelopment, could have significant impact on gliogenesis, but preserving or even enhancing neural differentiation. While the requirement is for removal of acetyl groups for the gliogenic switch to occur, it is possible that INHAT has a role in the dynamics of histone acetylation/deacetylation, leading to impairment of gliogenesis. This might be consistent with the delayed myelination reported in some SGS cases (Ko et al., 2013). Immunoprecipitation (IP) of SETBP1, SET, and ANP32 can be performed to determine the relationship dynamic between these proteins. IP-Mass Spec of histone marks could be done in SGS and SDD cells compared to controls to assess if INHAT activity is affected relative to SETBP1 levels. These marks could also be assessed in the differentiation process from neural progenitor cells to mature cell types such as neurons and oligodendrocytes to understand their effect. Transcription levels of genes influenced by these marks could be assessed to determine which cellular pathways and functions are affected.

## Protein Phosphatase 2A

Protein phosphatase 2A (PP2A) is a tumor suppressor gene that functions to slow cell proliferation; it removes phosphate groups from amino acids known to be important in mitosis (Mumby, 2007). PP2A is a serine/threonine phosphatase (Janssens et al., 2008), meaning that this complex of proteins cleaves phosphate groups from serine or threonine amino acids in specific peptide chains. The PP2A enzyme complex is composed of three different subunits: a scaffold subunit A, a regulatory subunit B, and a catalytic subunit C (Santa-Coloma, 2003; Janssens et al., 2008). Substrate specificity, tissue and cellular localization of the PP2A enzyme are determined by the association of specific subunits and presumably the 3D structure of the target molecule.

There are at least 300 substrates that have been demonstrated to be dephosphorylated by PP2A which may be tissue and cell type specific (Wlodarchak et al., 2016). While it is inherently difficult to determine targets of phosphatases, PP2A appears to remove the phosphate groups from a range of proteins involved in cell cycle regulation; from nuclear envelope proteins (Mehsen et al., 2018), or from signal transducers such as CJUN (Al-Murrani et al., 1999) and AKT (Alessi et al., 1996, 1997; Andrabi et al., 2007).

In a high affinity screen for molecules that bind PP2A, Li et al. (1995, 1996), identified I_1_PP2A and I_2_PP2A proteins in bovine kidney. I_1_PP2A was identified as ANP32A and I_2_PP2A was identified as SET (Li et al., 1995, 1996). ANP32 and SET family proteins could inhibit PP2A independently or working together (Santa-Coloma, 2003), and the SETBP1-SET binding site is notably different than the SET-PP2A binding site, so SETBP1 can interact with SET while it binds to PP2A (Figure 1; Minakuchi et al., 2001). The fact that SET and ANP32A are also both components of the INHAT complex and are capable of inhibiting PP2A is intriguing and presumably not random; however, the mechanisms governing regulation of these two complexes are unknown. It is possible that SET-ANP32A forms a complex that can both inhibit PP2A and form the INHAT complex, depending on stimuli received. SETBP1 could either act to sequester SET away from this complex, stabilize it so as to make it more likely to join this complex, or function as a platform to increase the likelihood of SET being part of one complex over another.

There is good evidence for a role for PP2A in neurodevelopment. For example, germline mutations in subunits of PP2A can cause neurodevelopmental disorders. Jordan’s syndrome (JR: *OMIM 616355*) is caused by mutations in *PPP2R5D*, *PPP2R5C*, *PPP2CA*, and *PPP2R1A* causing PP2A to be less active in cells and thus allowing phosphorylated target proteins to persist for too long (Reynhout et al., 2019), presumably enhancing proliferative effects. People with JR can have ventriculomegaly, epilepsy, intellectual delay, autism spectrum disorders as well as significant motor problems. Targeted investigation in model species also strongly support a role for PP2A in brain development. For example, mice with deletions of *Ppp2ca* are non-viable, where embryos show significant deficits in ectodermal tissue (giving rise to brain and skin), such as exencephaly and spina bifida (Panicker et al., 2020). A recent review of mouse models that modify or delete genes that code for specific *Pp2a* subunits further highlights the significant role of PP2A in brain (Reynhout and Janssens, 2019).

Schinzel–Giedion syndrome mutations in *SETBP1* likely lead to increased inhibition of PP2A *via* SET stabilization and therefore to persistence of phosphorylated targets of PP2A (similar to JR), while SDD *SETBP1* mutations will lead to loss of inhibition of PP2A and therefore too much cleavage of phosphate groups. We might expect these effects to be critical to cell cycle dynamics in neurodevelopment, especially since the timing of the cell cycle is so important during the expansion phase of neural progenitor cells (Ernst, 2016). For example, the cell cycle lengthens as neural progenitor cells expand (Caviness et al., 1995), so increasing the probability of mitosis by altering the activity of mitotic regulators such as phosphorylation state of the 308th and 473rd amino acid residues of AKT (Vanhaesebroeck and Alessi, 2000) could affect this timing and alter the total number of neural progenitor cells. We might then expect a hyperproliferative phenotype in SGS and lost proliferative capacity in SDD. Phosphorylation of direct targets of PP2A such as AKT, ERK, and GSK3-β could be measured in SDD and SGS cells relative to controls to assess the effect of SETBP1 protein levels in PP2A activity. Similarly, phosphorylation of PP2AC at its 307th amino acid residue, a known PP2A inactivation mark, could be used to assess PP2A activity.

## SET and DNA Nucleases (The “SET” Complex)

Granzymes induce a cell death pathway in blood cells activated in response to pathogens which can be transduced *via* an ER-anchored complex containing SET (Chowdhury and Lieberman, 2008). In this complex containing three DNA nucleases (NME1, TREX1, and APEX1), two chromatin modifiers (SET and ANP32A) and a DNA binding protein called HMGB2, granzymes released from other cells are thought to change the SET complex’s activity from a base excision repair function to a cell death function (Figure 1; Fan et al., 2003a; Chowdhury et al., 2006). When pathogens are present, they trigger granzyme release from cells which activates the SET containing complex in receiving cells to kill the cell. This is a mode of survival to stop viral replication. Under non-granzyme conditions, e.g., potentially in the central nervous system, the SET complex may function in DNA repair in response to oxidative damage (Leopoldino et al., 2012). Granzymes function to block the base excising repair function of the SET complex triggering more widespread DNA damage and apoptosis (Fan et al., 2003b), so the SET complex likely serves a DNA repair function in brain. Indeed, some members are critical for brain development (Bronstein et al., 2017; Dumitrache et al., 2018), though may be part of other complexes.

Might *SETBP1* mutations affect how SET interacts with NME1, APEX1, or HMGB2 to affect DNA in cell death or DNA repair pathways? If SETBP1 can sequester SET away from this complex, or affect how it gets cleaved it is possible that this important DNA nuclease complex gets altered in some way to affect DNA repair activities which are important in brain development, particularly in maintaining proliferative capacity of mitotically active neural progenitor cells (O’Driscoll and Jeggo, 2008). For example, HMGB2 is important in the neurogenic to gliogenic switch in developing neural stem cells (Bronstein et al., 2017), so affecting how SET associates with HMGB2 could affect some dynamics of cell fate switching in developing brain. We postulate that SGS mutations may lead to increased DNA nuclease activity which could cause too much DNA nicking and potentially lead to cell death. This could be through the stabilization of SET, allowing it to form more complexes with other SET complex members, or altering the probability of SET cleavage. SDD cases may suffer from reduced DNA repair capacity which may not be the opposite phenotype to the SGS cases (too much DNA nicking could lead to different phenotypes than too little DNA repair). This contrasts with SGS/SDD effects on PP2A where more or less phosphorylation of downstream PP2A targets could very likely lead to opposite affects (too much or too little p308-AKT, for example). To assess the effects that SETBP1/SET levels have in DNA integrity, BrdU and TUNEL assays can be performed as well as measurement of phosphorylated H2AX in SGS and SDD cells compared to controls.

## Set and Cell Cycle Control

Cell cycle control refers to the well-regulated process of cells as they progress through the cell cycle; that is, G1/0 phase, DNA synthesis (S) phase, G2 phase, and (M)itosis. An extensive array of proteins govern, for example, timing of phase shifts or checkpoints. These proteins were largely discovered during investigations of tumors in different tissues and many of these proteins are classified as oncogenes or tumor suppressors. The discovery of SET also fits into this pattern. SET was initially identified in the translocation fusion product SET-NUP214 in acute undifferentiated leukemia (von Lindern et al., 1992a) and SET is now known to bind to several regulators of the cell cycle. For example, SET binds to CDKN1A to modulate its inhibitory function on cyclin E to affect cell cycle progression through G1/S (Estanyol et al., 1999), and SET inhibits P53 by binding its C-terminal domain (Figure 1; Wang et al., 2016).

Cell cycle control is fundamental to brain development as it is to all tissues, though there are specific examples of its critical importance in brain through human mutation studies. For example, germline mutations in oncogenes and cell cycle regulators *PTEN* and *NF1* cause both a recognized tumor syndrome and vastly increased risk for autism spectrum disorders (Butler et al., 2005; Garg et al., 2013). Proteins such as P53 and CDKN1A can determine the extent of neural progenitor cell proliferation so their interaction with SET may be important in this process. Although SGS mutations in *SETBP1* have not been shown to increase the probability of SET to interact with CDKN1A, increased SETBP1 and SET levels have been shown to decrease p53 activity upon binding in neural cells, subsequently leading to DNA damage accumulation and parthanatos (Banfi et al., 2021). P53 may translocate to the nucleus and promote neuronal survival (Xavier et al., 2014) and p53 knockdown promotes neuronal differentiation (Marin Navarro et al., 2020). Neuronal differentiation could potentially be affected by altered SETBP1 levels in SDD and SGS, by the SET-induced effects in p53 activity.

Affecting these important players could alter timing of cell cycle exit or the expansion of undifferentiated neural progenitors. We expect that SGS mutations will lead to a more rapid cell cycle progression, enhancing proliferation, while SDD mutations could lead to decreased probability of mitosis. Cell cycle progression differences can be compared between SGS and SDD cells through DNA-labeled fluorescence activated cell sorting (FACS) and assessment of proportion of cells at G1/S/G2-M stage (where 2× DNA is observed in G2 phase compared to G0/G1 phase). Assessment of the interaction of SET/SETBP1 with other cell cycle control proteins such as cyclins and CDKs can be done through IP and immuno-blotting experiments.

## SETBP1 as a Transcription Factor and Epigenetic Regulator Independent From SET

Within its protein structure, SETBP1 contains three DNA binding domains, which are capable of binding to AT-rich regions on DNA (Piazza et al., 2018). Both wild-type and SGS SETBP1 proteins are likely able to bind to broad genomic regions containing the sequence “AAAATAA/T,” although SGS *SETBP1* mutations might do so at higher frequency due to its accumulation in the cell. There is evidence that SETBP1 bindS directly to DNA: chromatin immunoprecipitation studies suggest that SETBP1 can bind the promoters of *HOXA9*, *HOXA10*, and *RUNX1* in hematopoietic cells (Oakley et al., 2012; Vishwakarma et al., 2016). SETBP1 reportedly also binds to members of the KMT2A-COMPASS family HCF1, KMT2A, PHF8, and PHF6, which are responsible for the methylation of H3K4 at the promoters of developmentally regulated genes like the HOX gene clusters (Piazza et al., 2018). This may classify SETBP1 as an epigenetic regulator since COMPASS complexes can be recruited to chromatin by binding directly to DNA, by interacting with DNA-binding proteins or by interacting with modified histones (Piunti and Shilatifard, 2016; Lavery et al., 2020; Cenik and Shilatifard, 2021). It is not clear how or if SET may affect these particular interactions, but it cannot be ruled out. To elucidate if there is any effect of SET on SETBP1 capacity to act as a transcription factor, knockdown and/or overexpression of SET followed by assessment of SETBP1 DNA binding activity using Chromatin-Immunoprecipitation can be performed.

## Future Considerations

How *SETBP1* mutations affect developing brain is not known, but it seems reasonable to suggest that some of the molecular complexes in which SET is known to act in non-brain tissues might also be put to use in developing brain cells. There is already some recent evidence that this is indeed the case; for example, Banfi et al. (2021). suggest that *SETBP1* SGS mutations lead to SETBP1 accumulation in neurons and increased DNA damage which might suggest that association with SET and DNA nuclease activity may be important (Banfi et al., 2021). With the ability to rapidly make mouse models *via* CRISPR and to model human derived somatic cells from SGS/SDD cases in a neuronal context (Ernst, 2020), we expect rapid advances in the underlying mechanism of these two disorders. Both SGS and SDD mutation will lend themselves well to iPSC tissue modeling as monogenic diseases.

## Data Availability Statement

The original contributions presented in the study are included in the article/supplementary material, further inquiries can be directed to the corresponding author.

## Author Contributions

LA and CE wrote the manuscript. Both authors contributed to the article and approved the submitted version.

## Conflict of Interest

The authors declare that the research was conducted in the absence of any commercial or financial relationships that could be construed as a potential conflict of interest.

## Publisher’s Note

All claims expressed in this article are solely those of the authors and do not necessarily represent those of their affiliated organizations, or those of the publisher, the editors and the reviewers. Any product that may be evaluated in this article, or claim that may be made by its manufacturer, is not guaranteed or endorsed by the publisher.
